# Can the Nottingham Hip Fracture Score Predict Total Hip Replacement Versus Hemiarthroplasty Candidates?

**DOI:** 10.7759/cureus.94334

**Published:** 2025-10-11

**Authors:** David J Solomon, Rhiannon E Maunders, Abigail Adair, Andrew James

**Affiliations:** 1 Trauma and Orthopaedics, Ulster Hospital, Dundonald, GBR; 2 Trauma and Orthopaedics, Queen's University, Belfast, GBR

**Keywords:** hip fracture, neck of femur fractures, primary hemiarthroplasty, total hip replacement (thr), trauma

## Abstract

Background: The choice between total hip replacement (THR) and hemiarthroplasty in displaced intracapsular hip fractures remains debated. Procedure selection can be difficult given the current evidence. Using the Nottingham Hip Fracture Score (NHFS), we aimed to introduce objectivity into decision-making.

Methods: This is a retrospective single-centre review (2019-2023) of 1210 patients undergoing THR (n=228) or hemiarthroplasty (n=982). NHFS was calculated for all patients in this time period. Outcomes included one‑year mortality and discharge destination, with subgroup analysis of NHFS 4-6 and patients aged >80 with NHFS 4-6. Statistical analysis was completed using IBM SPSS Statistics for Windows, Version 28.0 (IBM Corp., Armonk, New York, United States).

Results: Hemiarthroplasty patients were older (82.2 vs. 74.6 years) and had higher mean NHFS (6.0 vs. 3.5) and higher one‑year mortality (36.2% vs. 3.5%). In the overall cohort, THR patients were more likely to be discharged home (65% vs. 39%), while hemiarthroplasty patients were more often discharged to institutional care. In the NHFS 4-6 subgroup, THR patients had lower mortality (3.4% vs. 29.6%; p<0.05) and more frequent home discharge (77.7% vs. 43.9%). In patients over 80 with NHFS 4-6, THR was associated with lower one-year mortality (4.2% vs. 13.8%) and a higher likelihood of independent living at home (75% vs. 60.9%) compared with hemiarthroplasty, although differences did not reach statistical significance.

Conclusion: Hemiarthroplasty patients are older and have higher mortality. Within NHFS 4-6 and in some circumstances, THR was associated with lower mortality and more favourable discharge outcomes. NHFS may complement the National Institute for Health and Care Excellence (NICE) guidance in identifying candidates most likely to benefit from THR vs. hip hemiarthroplasty.

## Introduction

Hip fractures represent one of the most significant challenges in modern orthopaedic practice, with profound clinical, social, and economic consequences. In the United Kingdom, more than 40% of trauma admissions are hip fractures each year, and with an ageing population, this number is expected to rise further [1]. Mortality following hip fracture remains high, with 30-day mortality rates of approximately 6-10% and one-year mortality approaching 30% [2]. Beyond survival, hip fractures frequently result in loss of independence, admission to long-term care facilities, and a major decline in quality of life. The economic burden is equally substantial, with annual costs to the National Health Service (NHS) exceeding £1.1 billion, driven by hospital admissions, surgical intervention, rehabilitation, and the long-term expense of institutional care [3]. 

Displaced intracapsular fractures of the femoral neck comprise a large proportion of these injuries and are almost universally treated surgically. The two dominant options are hemiarthroplasty and total hip replacement (THR). Hemiarthroplasty, in which only the femoral head is replaced, is technically less demanding, requires shorter operative time, and is associated with lower intraoperative blood loss. It has historically been the default operation for the majority of elderly and comorbid patients. In contrast, THR replaces both the acetabular and femoral components and offers the potential for superior long-term function, pain relief, and quality of life. However, it is a more complex operation associated with longer operative time, higher initial cost, and, historically, an increased risk of dislocation [4].

Guidance regarding which procedure should be performed has evolved. The National Institute for Health and Care Excellence (NICE) clinical guideline, updated in 2023, recommends that THR should be offered to patients with displaced intracapsular fractures who are independently mobile outdoors with no more than the use of a stick and who do not have a condition or comorbidity that makes the procedure unsuitable for them; also, they are expected to be able to carry out activities of daily living independently beyond two years [5]. Despite this guidance, national audit data consistently show that hemiarthroplasty remains the most frequently performed procedure, with more than 30,000 cases annually in the United Kingdom. Leaving the uptake of THR remains below guideline recommendations, reflecting challenges in patient selection, surgeon preference, resource constraints, and concerns regarding complications such as dislocation.

An important issue is the lack of a reliable, objective tool to guide decision-making at the patient level. Selection for THR is often subjective, based on the clinical impression of patient frailty, functional reserve, and comorbidity. This creates variation in practice and potential inequity in access to THR. The Nottingham Hip Fracture Score (NHFS), developed and validated in 2008, is a seven-parameter scoring system originally designed to predict 30-day mortality after hip fracture. It incorporates age, sex, comorbidities, cognitive status, haemoglobin, presence of malignancy, and pre-injury residence. Scores range from 0 to 10, with higher scores indicating high mortality risk. The NHFS is validated across multiple populations and is widely used in clinical audit and research [6]. Patients with low NHFS scores are generally young, fit, and obvious candidates for THR, while those with high scores are frail and often best served with hemiarthroplasty. However, a substantial proportion of patients fall into an intermediate range of NHFS 4-6, representing a "grey zone" where the optimal surgical procedure is uncertain and the decision between THR and hemiarthroplasty is often most challenging in this group.

This study, therefore, aimed to evaluate outcomes of THR and hemiarthroplasty in displaced intracapsular fractures, stratified by NHFS. Specifically, we examined outcomes at the Ulster Hospital Dundonald (UHD) over five years. This study looks at the full cohort, the intermediate-risk subgroup (NHFS 4-6), and the >80-year-olds with an NHFS of 4-6. We hypothesised that NHFS, when used alongside NICE guidance, may help identify patients most likely to benefit from THR, thereby introducing greater objectivity into operative selection.

## Materials and methods

A retrospective, single-centre cohort study was conducted at the UHD, Northern Ireland, between January 2019 and December 2023.

Inclusion criteria

All patients aged ≥60 years presenting with a displaced intracapsular hip fracture and treated surgically with either THR or hemiarthroplasty were included.

Exclusion criteria

All patients with pathological fractures, polytrauma, periprosthetic fractures, non-displaced fractures managed non-operatively, and incomplete NHFS data were excluded.

Data collection

Electronic health records, operative logs, and discharge summaries were reviewed. Variables collected included age, sex, comorbidities, American Society of Anesthesiologists (ASA) grade, Abbreviated Mental Test Score (AMTS), haemoglobin on admission, malignancy history, pre-fracture residence status, operative procedure, and discharge destination. NHFS was calculated retrospectively for each patient using the original seven parameters [6].

Outcomes

The primary outcome was one‑year all-cause mortality, verified using hospital records. Secondary outcomes included discharge destination at one year (home, hospital transfer, institutional care) and subgroup analysis of patients with NHFS scores 4-6 and also the subgroup >80 years old with NHFS 4-6.

Statistical analysis

Continuous variables were reported as means with standard deviations and compared using independent t‑tests. Categorical variables were reported as proportions and compared using chi‑squared tests. Statistical significance was set at p<0.05. Analysis was conducted using IBM SPSS Statistics for Windows, Version 28.0 (IBM Corp., Armonk, New York, United States).

## Results

Full cohort

A total of 1210 patients met the inclusion criteria, of whom 982 (81.2%) underwent hemiarthroplasty and 228 (18.8%) underwent THR. Baseline demographics are summarised in Table 1. Patients treated with hemiarthroplasty were significantly older (mean age 82.2 vs. 74.6 years) and had higher ASA grades and NHFS scores (6.0 vs. 3.5). One-year mortality in the full cohort was substantially higher following hemiarthroplasty compared with THR (36.2% vs. 3.5%; p<0.001). Twelve-month outcomes also differed between groups (Table 2). Around 89.9% of THR patients remained living at home compared with only 39.2% of hemiarthroplasty patients. These differences were statistically significant (p<0.001).

**Table 1:** Baseline demographics Table summarising the baseline demographics of all intracapsular neck of femur fracture patients between 2019 and 2023. THR: total hip replacement; ASA: American Society of Anesthesiologists; NHFS: Nottingham Hip Fracture Score

Variable	Hemiarthroplasty	THR
n	982	228
Mean age (SD)	82.22 (8.81)	74.64 (9.55)
Mean ASA (SD)	3.18 (0.6)	3.0 (9.93)
Mean NHFS (SD)	6.03 (1.34)	3.5 (1.35)
1‑year mortality (%)	36.15	3.51

**Table 2:** Living status at 12 months Table summarising the residence at 12 months for all patients with intracapsular neck of femur fractures between 2019 and 2023. Values compared using the chi-squared test (χ²=63.8; df=1; p<0.001). Categorical variables reported as counts and percentages. THR: total hip replacement

Status at 12 months	Hemiarthroplasty (n=982)	Hemiarthroplasty %	THR (n=228)	THR %
Home/independent	385	39.2	205	89.9
Residential/nursing	243	24.7	12	5.3
Deceased	355	36.1	8	3.5
Other/unknown	-	-	3	1.3

Subgroup analysis: NHFS 4-6

A total of 365 patients were classified with an NHFS between 4 and 6 (hemiarthroplasty n=196; THR n=169). At 12 months, mortality was significantly lower in the THR group compared with hemiarthroplasty (2.4% vs. 15.8%; p=0.0001). At one year, the majority of THR patients were independent at home (79%) compared with less than half of hemiarthroplasty patients (44%). A small number of cases in both groups could not be categorised due to incomplete follow-up records (Other/Unknown). These findings indicate that in the intermediate-risk NHFS group, THR was associated with both superior survival and a greater likelihood of returning to independent living (Table 3).

**Table 3:** Subgroup NHFS 4-6 Table showing analysis of subgroup NHFS 4-6 12-month residence status and mortality data. Values compared using the chi-squared test (χ²=15.3; df=1; p=0.00019). NHFS: Nottingham Hip Fracture Score; THR: total hip replacement

Residential status at 12 months: NHFS 4-6	THR n	THR %	Hemiarthroplasty n	Hemiarthroplasty %
Home/independent	151	89.3	122	62.2
Residential care	1	0.6	4	2
Nursing care	1	0.6	0	0
Hospital/transfer	0	0	0	0
Deceased	3	1.8	25	12.8
Other/unknown	13	7.7	45	23

Subgroup analysis: >80 years old with NHFS 4-6

In the subgroup of patients aged over 80 years with NHFS scores 4-6, outcomes were more favourable for those undergoing THR compared with hemiarthroplasty, although numbers in the THR group were limited. At 12 months, three-quarters of THR patients (75%) were living independently at home, compared with 60.9% of hemiarthroplasty patients. Mortality was also lower after THR (4.2% vs. 13.8%), although this difference did not reach statistical significance (p=0.35).

**Table 4:** Twelve-month mortality and living status in patients >80 years with NHFS 4-6 Table showing the mortality and residence status of patients at 12 months. Values compared using the chi-squared test (χ²=0.86; df=1; p=0.35). THR: total hip replacement

Outcome	THR (n=24)	%	Hemiarthroplasty (n=87)	%
Home/independent	18	75	53	60.9
Residential care	0	0	2	2.3
Nursing care	0	0	0	0
Hospital/transfer	1	4.2	0	0
Deceased	1	4.2	12	13.8
Other/unknown	4	16.7	20	23

## Discussion

This study demonstrates that NHFS may help guide operative choice in displaced intracapsular fractures. While hemiarthroplasty patients were older and higher risk overall, THR was associated with lower mortality and more favourable 12-month outcomes, particularly in the NHFS 4-6 subgroup. This suggests that NHFS could be used in conjunction with existing NICE guidance to better stratify candidates for THR.

From a health economics perspective, hip fractures cost the NHS approximately £1.1 billion annually, with the average cost per patient exceeding £14,000. Institutional care is a major driver, costing £600-800 per week. Higher rates of home discharge among THR patients imply potential downstream cost savings that may offset the increased operative complexity and implant cost of THR [7]. 

Our results complement existing national and international data by highlighting the survival and independence benefits of THR, particularly in patients with intermediate NHFS scores. The present findings, therefore, suggest that the benefits of THR may extend beyond the narrow selection criteria currently applied in routine practice. Recent trials and meta-analyses, including the HEALTH trial, have demonstrated functional advantages for THR while also noting an increased risk of dislocation [8]. Taken together, this data supports a more objective approach to procedure selection, incorporating tools such as the NHFS alongside clinical judgement and NICE guidance. 

Dislocation remains the major complication of THR in the fracture setting. However, the introduction of dual‑mobility cups and bipolar prostheses has significantly reduced this risk, particularly in elderly and high‑risk cohorts. Several large registry studies have demonstrated dislocation rates as low as 1-2% when dual‑mobility implants are used, compared with 5-7% for conventional THR [9]. This suggests that the use of such implants may make THR a safer option in intermediate‑risk patients identified by NHFS.

Surgical approach is another determinant of outcome. THR is typically performed using a posterior approach, which preserves abductor function and facilitates gait recovery, although it may carry a slightly higher dislocation risk. Hemiarthroplasty is more often performed using an anterolateral approach, which reduces dislocation but involves partial abductor detachment, potentially impairing long‑term mobility. These differences may contribute to the superior functional recovery often reported with THR [10].

Another important factor is the restoration of biomechanics. Historically, leg length discrepancy and offset mal‑restoration were common concerns with THR. However, advances in preoperative templating and intraoperative measuring tools have substantially reduced these risks. Studies demonstrate that modern templating achieves leg length accuracy within 5 mm in the majority of cases, reducing one of the major functional drawbacks of THR [11].

Surgeon experience also influences outcomes. Evidence indicates that THR performed by senior arthroplasty surgeons is associated with lower complication rates and better functional outcomes than when performed by junior or non‑arthroplasty-trained surgeons [12]. By contrast, hemiarthroplasty is frequently performed by registrars or non‑specialists, which may partly explain differences in outcomes between the two procedures. 

Implant choice in hemiarthroplasty is also important. While traditional monoblock implants such as the Exeter Trauma Stem (ETS) remain widely used, registry data suggest that modular or bipolar designs such as the Unitrax may reduce acetabular wear and improve long‑term function [13,14]. If hemiarthroplasty remains the predominant procedure, greater attention should be paid to optimising implant selection to restore leg length and offset to maximise patient outcomes.

The role of NHFS in this context is notable. While originally validated for mortality prediction, our results suggest NHFS may also help identify intermediate‑risk patients (scores 4-6) who may benefit most from THR. This practical application could support more nuanced decision-making in real-world practice, where hemiarthroplasty remains more common despite accumulating comparative operative procedures.

Strengths of this study include a large sample size, consecutive patient inclusion, and pragmatic outcomes of mortality and residence at the one-year follow-up.

This study has some limitations; mainly, its retrospective single-centre design limits generalisability. The sample sizes reduced the power in subgroup analyses, especially among patients over 80 years with intermediate NHFS scores. Several clinically important variables were not analysed, including time to surgery, intraoperative complications, reoperation rates, and validated functional outcomes. This means that results are restricted to mortality and residence status. NHFS was designed as a mortality prediction, not a surgical choice; therefore, at present, there is limited evidence for predicting surgical suitability.

These issues highlight the need for larger, multicentre prospective studies with complete datasets to validate these findings. 

## Conclusions

The NHFS differentiates patients undergoing THR and hemiarthroplasty. Within the intermediate‑risk NHFS 4-6 subgroup, THR was associated with significantly lower one‑year mortality and higher rates of positive 12-month living arrangement compared with hemiarthroplasty. These findings suggest NHFS may help identify patients in this "grey zone" who could benefit from THR, complementing NICE guidance and supporting pragmatic clinical decision‑making. While THR carries greater operative complexity and risk of dislocation, advances in implant design, templating, and surgical expertise have reduced these risks. The potential benefits in survival, function, and resource utilisation warrant serious consideration in intermediate‑risk patients. Future prospective multicentre studies and cost‑effectiveness analyses are required to further validate this role for NHFS in guiding arthroplasty choice.
